# Variance-adjusted Mahalanobis (VAM): a fast and accurate method for cell-specific gene set scoring

**DOI:** 10.1093/nar/gkaa582

**Published:** 2020-07-07

**Authors:** Hildreth Robert Frost

**Affiliations:** Department of Biomedical Data Science, Geisel School of Medicine, Dartmouth College, Hanover, NH 03755, USA

## Abstract

Statistical analysis of single cell RNA-sequencing (scRNA-seq) data is hindered by high levels of technical noise and inflated zero counts. One promising approach for addressing these challenges is gene set testing, or pathway analysis, which can mitigate sparsity and noise, and improve interpretation and power, by aggregating expression data to the pathway level. Unfortunately, methods optimized for bulk transcriptomics perform poorly on scRNA-seq data and progress on single cell-specific techniques has been limited. Importantly, no existing methods support cell-level gene set inference. To address this challenge, we developed a new gene set testing method, Variance-adjusted Mahalanobis (VAM), that integrates with the Seurat framework and can accommodate the technical noise, sparsity and large sample sizes characteristic of scRNA-seq data. The VAM method computes cell-specific pathway scores to transform a cell-by-gene matrix into a cell-by-pathway matrix that can be used for both data visualization and statistical enrichment analysis. Because the distribution of these scores under the null of uncorrelated technical noise has an accurate gamma approximation, both population and cell-level inference is supported. As demonstrated using simulated and real scRNA-seq data, the VAM method provides superior classification accuracy at a lower computation cost relative to existing single sample gene set testing approaches.

## INTRODUCTION

### Single cell transcriptomics

Despite the diversity of cell types and states present in multicellular tissues, high-throughput genome-wide profiling has, until recently, been limited to assays performed on bulk tissue samples. For bulk tissue assays, the measured values reflects the average across a large number of cells and, when significant heterogeneity exists, only approximate the true biological state of the tissue. To address the shortcomings of bulk tissue analysis, researchers have developed a range of techniques for the genome-wide profiling of individual cells (1,2) with single cell RNA sequencing (scRNA-seq) (3) generating particular scientific interest due to the rapid development of the underlying laboratory techniques, which can now cost-effectively quantify genome-wide transcript abundance for thousands to tens-of-thousands of cells. Single cell genomic assays, in combination with techniques that infer transcription rates (4), spatial information (5) or temporal dynamics (6,7), provide scientists with a detailed picture of cellular biology. Such cell-level genomic resolution is especially important for the study of tissues whose structure and function is defined by complex interactions between multiple distinct cell types that can occupy a range of phenotypic states, e.g. the tumor microenvironment (8,9), immune cells (10,11), and the brain (12).

### Single cell analysis challenges

Although single cell data provides unprecedented insights into the structure and function of complex tissues and cell populations, technical and biological limitations make statistical analysis challenging (13). Single cell methods analyze very small amounts of genomic material, leading to significant amplification bias and inflated zero counts relative to bulk tissue assays (14). Single cell-specific approaches for quality control, normalization and statistical analysis (e.g. zero-inflated models) only partially address these challenges (15,16). In addition to the challenges of increased noise and missing data, important biological differences exist between bulk tissue and single cell data. As the average over a large number of cells, bulk tissue measurements are typically unimodal and, in many cases, approximately normally distributed. In contrast, single cell data sets reflect a heterogeneous mixture of cell types and cell states resulting in multi-modal and non-normal distributions (14). The diverse mixture of cell types and states found in complex tissues also leads to significant differences in gene expression patterns between bulk tissue and single cell data. As evidenced by projects such as the Human Protein Atlas (HPA) (17), gene activity measured on bulk tissue samples can differ substantially from the activity occurring within the cell subpopulations comprising the tissue. Collectively, the distributional differences between single cell and bulk tissue genomic data make it challenging to successfully analyze single cell expression data using methods originally developed for bulk tissue, which were optimized for non-sparse gene expression data with lower levels of technical noise and moderate sample size.

### Gene set testing of single cell data

Although high-dimensional genomic data provides a molecular-level lens on biological systems, the gain in fidelity obtained by testing thousands of genomic variables comes at the price of impaired interpretation, loss of power due to multiple hypothesis correction and poor reproducibility (18–21). To help address these challenges for bulk tissue data, researchers developed gene set testing, or pathway analysis, methods (21,22). Gene set testing is an effective hypothesis aggregation technique that lets researchers step back from the level of individual genomic variables and explore associations for biologically meaningful groups of genes. By focusing the analysis on a small number of functional gene sets, gene set testing can substantially improve power, interpretation and replication relative to an analysis focused on individual genomic variables (18–21). The benefits that gene set-based hypothesis aggregation offers for the analysis of bulk tissue data are even more pronounced for single cell data given increased technical variance and inflated zero counts.

Gene set testing methods can be categorized according to whether they support supervised or unsupervised analyzes (i.e. test for association with a specific clinical endpoint or test for enrichment in the variance structure of the data), whether they provide results for each sample or for an entire population, whether they test a self-contained or competitive null hypothesis (i.e. the *H*_0_ that none of the genes in the set has an association with the outcome or the *H*_0_ that the genes in the set are not more associated with the outcome than genes not in the set) and whether they test each gene set separately (uniset) or jointly evaluate all sets in a collection (multiset). In this paper, we focus on single sample gene set testing methods, i.e. those that compute a cell-specific statistic for each analyzed gene set to transform a cell-by-gene scRNA-seq matrix into a sample-by-pathway matrix. This class of techniques is of particular interest because the cell-level pathway scores can be leveraged for both exploratory data visualization, e.g. shading of cells in a reduced dimensional plot according to inferred pathway activity, as well as the full range of population-level statistical gene set tests, i.e. supervised or unsupervised tests of either the uniset or multiset flavor.

Existing single sample gene set testing methods can be grouped into three general categories: random walk methods, principal component analysis (PCA)-based methods and z-scoring methods. Random walk methods (e.g. GSVA (23) and ssGSEA (24)) generate sample-level pathway scores using a Kolmogorov–Smirnov (KS) like random walk statistic computed on the gene ranks within each sample, often following some form of gene standardization across the samples. AUCell (25) also generates cell-level gene set scores based on gene ranks within each cell but uses a more simplistic approach than GSVA or ssGSEA that does not take into account gene set size or the distribution of gene expression values across all cells in the data set. Specifically, AUCell computes the proportion of the top-ranked genes are also members of a given gene set, where the number of top-ranked genes to consider is determined by the user. PCA-based methods (e.g. PAGODA (26) and PLAGE (27)) perform a PCA on the expression data for each pathway and use the projection of each sample onto the first PC as a sample-level pathway score. *Z*-scoring methods (e.g. technique of Lee *et al.* (28), scSVA (29) and Vision (30)) generate pathway scores based on the standardized mean expression of pathway genes within each sample. While these methods have proven effective for the analysis of bulk expression data, with GSVA and ssGSEA among the most popular techniques, the application of these methods to scRNA-seq data is limited by three main factors: poor classification performance in the presence of sparsity and technical noise, lack of inference support on the single cell level, and high computational cost (esp. for the random walk methods when the number of samples/cells is large).

GSVA, ssGSEA, PLAGE and the Lee *et al.z*-scoring methods were all developed for the analysis of bulk gene expression data and were therefore optimized for, and evaluated on, non-sparse data with moderate levels of technical noise. Although AUCell, scSVA and Vision are all targeted at single cell expression data, they make no special provision for the statistical characteristics of single cell data such as sparsity and elevated noise. As we demonstrate through simulation studies later in the manuscript, these methods all have poor classification performance relative to the VAM technique on sparse and noisy data, i.e. they are not able to effectively identify cells whose transcriptomic profile is enriched for specific pathways. In contrast to the other existing single sample methods, PAGODA was designed for single cell analysis and specifically addresses the scRNA-seq features of sparsity and technical noise. In the case of PAGODA, however, the primary focus is an unsupervised and population-level analysis; the generation of sample-level scores is a secondary output which lacks inference support. Relative to the random walk and *z*-scoring approaches, the class of PCA-based methods, which includes PAGODA, is particularly poor at identifying cells with elevated expression of specific pathways in simulated data sets.

Although the pathway scores generated by the *z*-scoring methods should have a standard normal distribution when the expression data follows an uncorrelated multivariate normal distribution, this distributional assumption does not hold for sparse scRNA-seq data. Neither the random walk nor the PCA-based method generate scores with a well characterized null distribution. While the lack of a null distribution does not prevent the cell-specific scores generated by these techniques from being used for visualization or as predictors in regression models, it does preclude cell-level inference and the use of scores as dependent variables in parametric models.

Given experimental and cost constraints, most bulk gene expression data sets have sample sizes in the hundreds; bulk data sets with more than one thousand samples are rare. Single cell data sets, by contrast, typically profile thousands of cells and data sets containing tens-of-thousands to hundreds-of-thousands of cells are becoming increasingly common. These large sample sizes make computational cost an important factor, especially for techniques that are used in an exploratory and interactive context. Relative to the VAM approach, all of the existing single sample methods have significantly worse computational performance on even small (2000 cells, 500 genes) data sets. For very large scRNA-seq data sets (i.e. 100 000+ cells), the use of methods like GSVA and ssGSEA will be impractical for many users.

To support the gene set analysis of scRNA-seq data and address the limitations of existing gene set testing methods, we developed the VAM technique, which was specifically designed for the analysis of large, noisy and sparse transcriptomic data. In the remainder of the paper, we provide an overview of the VAM method, detail its statistical characteristics, and illustrate the comparative benefits of VAM via both simulation studies and real data analyses. An R package implementing the VAM method and several example vignettes are available at http://www.dartmouth.edu/∼hrfrost/VAM.

## MATERIALS AND METHODS

### Variance-adjusted Mahalanobis (VAM)

The VAM method generates cell-specific gene set scores from scRNA-seq data using a variation of the classic Mahalanobis multivariate distance measure (31). VAM takes as input two matrices:

- $\mathbf {X}$: *n* × *p* matrix that holds the positive normalized counts for *p* genes in *n* cells as measured via scRNA-seq. As detailed in the *VAM-Seurat integration* Section below, VAM provides direct support for both Seurat (32) normalization techniques: log-normalization (i.e. log of 1 plus the unnormalized count divided by an appropriate scale factor for the cell) and the SCTransform method (33). Other scale factor-based normalization techniques that are equivalent to Seurat log-normalization (e.g. normalization supported by the Scater framework (15)) can also be used.
- $\mathbf {A}$: *m* × *p* matrix that represents the annotation of *p* genes to *m* gene sets as defined by a collection from a repository like the Molecular Signatures Database (MSigDB) (34) (*a*_*i*, *j*_ = 1 if gene *j* belongs to gene set *i*).

VAM generates as output one matrix:

- $\mathbf {S}$: *n* × *m* matrix that holds the cell-specific scores for each of the *m* gene sets defined in $\mathbf {A}$.

Given $\mathbf {X}$ and $\mathbf {A}$, VAM computes $\mathbf {S}$ using the following steps:

1. **Estimate technical variances**: Let $\hat{\boldsymbol{\sigma }}_{\text{tech}}^2$ be a length *p* vector holding the technical component of the sample variance of each gene in $\mathbf {X}$. For the VAM-Seurat integration, two approaches are supported for computing $\hat{\boldsymbol{\sigma }}_{\text{tech}}^2$ depending on whether log-normalization or SCTransform is employed (see the *VAM-Seurat integration* Section below for details). Similar variance decomposition approaches are supported by other scRNA-seq normalization pipelines (e.g. Scater (15)). VAM can also be used under the assumption that the observed marginal variance of each gene is entirely technical. In this case, $\hat{\boldsymbol{\sigma }}_{\text{tech}}^2$ is simply estimated by the sample variances of each gene in $\mathbf {X}$.
2. **Compute modified Mahalanobis distances**: Let $\mathbf {M}$ be an *n* × *m* matrix of squared values of a modified Mahalanobis distance. Each column *k* of $\mathbf {M}$, which holds the cell-specific squared distances for gene set *k*, is calculated as:
  (1) $$\begin{equation*} \mathbf {M}[,k] = \mathbf {X}_k^T (\mathbf {I}_{g} \hat{\boldsymbol{\sigma }}_{g,\text{tech}}^2)^{-1} \mathbf {X}_k \end{equation*}$$
  where *g* is the size of gene set *k*, $\mathbf {X}_k$ is a *n* × *g* matrix containing the *g* columns of $\mathbf {X}$ corresponding to the members of set *k*, $\mathbf {I}_{g}$ is a *g* × *g* identity matrix, and $\hat{\boldsymbol{\sigma }}_{g,\text{tech}}^2$ holds the elements of $\hat{\boldsymbol{\sigma }}_{\text{tech}}^2$ corresponding to the *g* genes in set *k*.
3. **Compute modified Mahalanobis distances on permuted $\mathbf {X}$**: To capture the distribution of the squared modified Mahalanobis distances under the *H*_0_ that the normalized expression values in $\mathbf {X}$ are uncorrelated with only technical variance, the distances are recomputed on a version of $\mathbf {X}$ where the row labels of each column are randomly permuted. Let $\mathbf {X_p}$ represent the row-permuted version $\mathbf {X}$ and let $\mathbf {M_p}$ be the *n* × *m* matrix that holds the squared modified Mahalanobis distances computed on $\mathbf {X_p}$ according to (1).
4. **Fit gamma distribution to each column of $\mathbf {M_p}$**: A separate gamma distribution is fit using the method of maximum likelihood (as implemented by the *fitdistr()* function in the MASS R package (35)) to the non-zero elements in each column of $\mathbf {M_p}$. Let $\hat{\alpha }_k$ and $\hat{\beta }_k, k \in {1,...,m}$ represent the gamma shape and rate parameters estimated for gene set *k* using this procedure. As detailed in the *Statistical properties of VAM* Section below, the normal χ^2^ approximation for standard squared Mahalanobis distances does not hold for the values generated according to (1), however, the null distribution of these values can be well characterized by a gamma estimated on each column of $\mathbf {M_p}$. Note that if computational efficiency is a major concern, the gamma distributions can be fit directly on $\mathbf {M}$ to avoid the cost of generating $\mathbf {X_p}$ and $\mathbf {M_p}$; this will impact the power to detect deviations from *H*_0_ but will not inflate the type I error rate.
5. **Use gamma cumulative distribution function (CDF) to compute cell-specific scores**: The cell-specific gene set scores are set to the gamma CDF value for each element of $\mathbf {M}$. Specifically, each column *k* of $\mathbf {S}$, which holds the cell-specific scores for gene set *k*, is calculated as:
  (2) $$\begin{equation*} \mathbf {S}[,k] = F_{\gamma (\hat{\alpha }_k, \hat{\beta }_k)}(\mathbf {M_p}[,k]) \end{equation*}$$
  where $F_{\gamma (\hat{\alpha }_k, \hat{\beta }_k)}()$ is the CDF for the gamma distribution with shape $\hat{\alpha }_k$ and rate $\hat{\beta }_k$. Under the *H*_0_ of uncorrelated technical noise, valid *P*-values can be generated by subtracting the elements of $\mathbf {S}$ from 1. The *Statistical properties of VAM* Section explores the statistical properties of the elements of $\mathbf {M}$ and inference using *P*-values generated via $\boldsymbol{1}-\mathbf {S}$ in greater detail.

The use of $F_{\gamma (\hat{\alpha }_k, \hat{\beta }_k)}()$ to generate the elements of $\mathbf {S}$ has several important benefits in addition to support for cell-level inference. First, it transforms the squared modified Mahalanobis distances for gene sets of different sizes into a common scale, which is important if values in $\mathbf {S}$ are used together in statistical models, e.g. as regression predictors. Second, it generates a statistic that is bound between 0 and 1 and is robust to very large expression values, i.e., the CDF converges quickly to 1 as the squared distances increase. Such robustness is particularly important for the analysis of noisy scRNA-seq data; many existing scRNA-seq analysis methods such as SCTransform artificially clip normalized data to eliminate extreme values. Lastly, the fact that the distribution of values is often bimodal with most values close to 0 or 1 improves the utility of $\mathbf {S}$ for both visualization and statistical modeling.

### Comparison of VAM and the standard Mahalanobis distance

For the scenario represented by (1), the squared Mahalanobis distance is normally defined as:

(3) $$\begin{equation*} \mathbf {M}[,k] = (\mathbf {X}_k-\bar{\mathbf {X}}_k)^T \hat{\boldsymbol{\Sigma }}_{k}^{-1} (\mathbf {X}_k-\bar{\mathbf {X}}_k) \end{equation*}$$

where $\bar{\mathbf {X}}_k$ is a matrix whose rows contain the mean values of the columns of $\mathbf {X}_k$ and $\hat{\boldsymbol{\Sigma }}_{k}$ is the estimated sample covariance matrix for $\mathbf {X}_k$. There are two important differences between the modified Mahalanobis distance in (1) and the standard Mahalanobis distance in (3):

1. The standard Mahalanobis distance uses the full sample covariance matrix whereas the modified Mahalanobis distance accounts for just the technical variance of each gene and ignores covariances.
2. The standard Mahalanobis measure computes the distances from the multivariate mean whereas the modified Mahalanobis distance computes distances from the origin.

A key feature of the VAM method, and the basis for the ‘variance-adjusted’ portion of the name, is the use of $\mathbf {I}_g \hat{\boldsymbol{\sigma }}_{g,\text{tech}}^2$ instead of the sample covariance matrix included in the typical Mahalanobis formulation. The practical impact of this change is that deviations in directions of large estimated technical variance are discounted (i.e. larger deviations are expected due to the higher variance) but deviations in directions of large biological variation (or covariance) are not discounted (i.e. these deviations are not expected if the variation in expression is purely technical).

Use of the origin instead of the multivariate mean in (1) generates a more biologically meaningful distance measure for scRNA-seq data. With the standard Mahalanobis distance, it is possible for samples whose elements are all above the mean, all below the mean or a mixture of above and below to have the exact same distance value. Computing distances from the origin for positive data eliminates this ambiguity: larger distances correspond to larger positive sample values, i.e. elevated gene expression in the cell, and a distance of 0 corresponds to lack of expression in all genes. Measuring distances from the origin will also assign more extreme values to sets whose members show coordinated expression. When distances are measured from the multivariate mean, it is not possible distinguish between sets with a mixture of up and down-regulated genes and sets whose members show coordinated expression. Prioritizing coordinated expression is advantageous since such pathways are usually more biologically interesting. As a simple example, imagine a two gene set with mean (1,1) and identity covariance matrix. For this set, cells with expression values of (0,0), (2,0), (0,2) and (2,2) all have the same squared Mahalanobis distance of 2 when distances are measured from the multivariate mean. By contrast, the squared distance from the origin for these cells is 0, 4, 4 and 8, which better reflects the combined expression of these genes. It should be noted that the difference between the mean and origin will usually be minor for scRNA-seq data since most genes will have mean values very close to 0.

### Statistical properties of VAM

If the values in $\mathbf {X}_k$ follow a multivariate normal distribution, the squared Mahalanobis distances computing according to the standard definition in (3) can be approximated by a χ^2^ distribution with *g* degrees-of-freedom, where *g* is the size of gene set *k*. If $\bar{\mathbf {X}}_k$ is replaced by the $\boldsymbol{0}$ vector in (3), the resulting squared distances are instead approximated by a non-central χ^2^ distribution with *g* degrees-of-freedom and non-centrality parameter $\bar{\mathbf {X}}_k^T \hat{\boldsymbol{\Sigma }}_{k}^{-1} \bar{\mathbf {X}}_k$.

**Figure 1.**
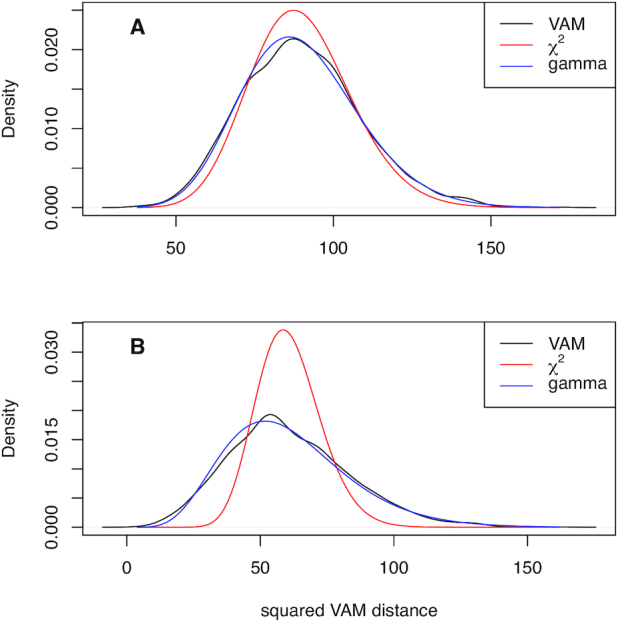
Distribution of squared modified Mahalanobis distances computed using (1) on scRNA-seq data simulated under the *H*_0_ of uncorrelated technical noise as detailed in the SI Methods. The densities of the non-central χ^2^ approximation and estimated gamma distribution are also plotted. (**A**) Density estimates for data with a simulated sparsity of 0.5. (**B**) Density estimates for data with a simulated sparsity of 0.8.

The modified squared Mahalanobis measure used by VAM and defined in (1) can also be approximated by a non-central χ^2^ distribution under the *H*_0_ of uncorrelated technical noise if the data in $\mathbf {X}_k$ is not too sparse, i.e. ∼50% or fewer of the elements are zero, and the non-zero values in $\mathbf {X}_k$ have an approximately normal distribution. Figure 1 illustrates the density estimate for values computed using (1) on scRNA-seq data simulated under the *H*_0_ of uncorrelated technical noise for sparsity values of both 0.5 and 0.8 (see the SI Methods for more details on the simulation model, which assumes a log-normal distribution for the non-zero elements in $\mathbf {X}_k$). Figure 1 also includes the density for the non-central χ^2^ distribution with the appropriate degrees-of-freedom and non-centrality parameter. As shown in this figure, the non-central χ^2^ distribution provides an accurate approximation for a sparsity of 0.5 (panel A), but overestimates the mean and significantly underestimates the variance of the squared distances when the sparsity increases to 0.8 (panel B).

Given the poor fit of a non-central χ^2^ distribution for realistic sparsity levels, we instead model the null distribution of elements in $\mathbf {M}$ by a gamma distribution whose parameters are estimated via maximum likelihood as described above. As shown in Figure 1, the estimated gamma distribution provides a very good fit for the observed squared modified Mahalanobis distances at both the 0.5 and 0.8 sparsity levels. The type I error control and power provided by the estimated gamma distribution is detailed in the *Type I error control and power* Section below.

### Comparison methods

For comparative evaluation of the VAM method on both simulated and real scRNA-seq data, we used methods from each of the existing categories of single sample gene set testing methods. For the random walk category, we used both GSVA (23) and ssGSEA (24) given the popularity of these two techniques, for the class of *z*-scoring methods, we used the technique of Lee *et al.* (28), and, for the class of PCA-based methods we used PLAGE (27). For all of these comparison methods, the implementations available in the GSVA R package were employed. Unless otherwise noted, analyses were performed using default values for method parameters.

## RESULTS AND DISCUSSION

### Type I error control and power

Type I error control was assessed using scRNA-seq data simulated according to the process detailed in the SI Methods with the technical variances set to the sample variance of the simulated genes. In particular, the non-zero counts were simulated according to a log-normal distribution estimated from real scRNA-seq data (see Supplementary Figure S1). The VAM method was applied to a set comprised by 50 randomly selected genes. The type I error rate at an α = 0.05 for 10 simulated scRNA-seq data sets (2000 *P*-values per data set for 20 000 total hypothesis tests) was 0.048. To assess power, a random group of 50 genes were given inflated log-normal values for the first 50 cells with the mean value ranging from 0.7 to 1.7 (the non-inflated mean was 0.642 to align with the PBMC data). For each inflated mean value, 10 data sets were simulated and power was computed on the 50 non-null cells for a total of 500 hypothesis tests. The estimated power values ranged from 0.11 for an inflated mean of 0.7 to 0.99 for an inflated mean of 1.7 (this power curve is illustrated in Supplementary Figure S2).

### Classification performance

**Figure 2.**
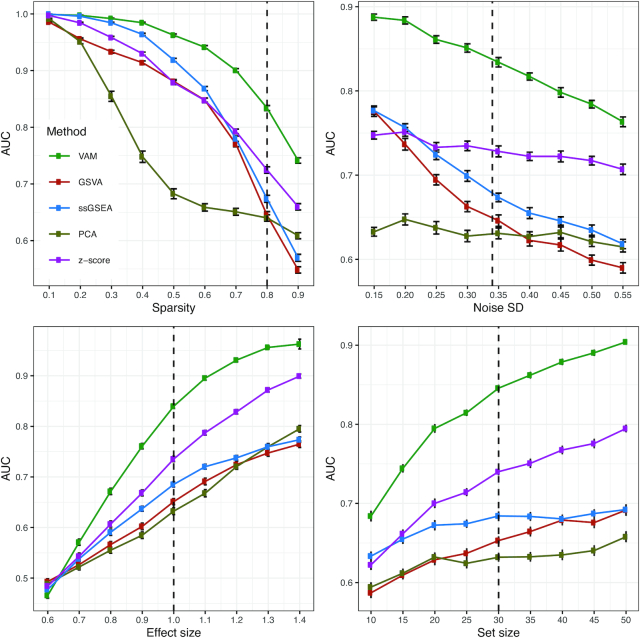
Classification performance of VAM, GSVA, ssGSEA and representative z-scoring and PCA-based methods on scRNA-seq data simulated using the procedure detailed in the SI Methods. Each panel illustrates the relationship between the area under the receiver operating characteristic curve (AUC) and one of the simulation parameters. The vertical dotted lines mark the default parameter value used in the other panels. Error bars represent the standard error of the mean.

To compare the performance of VAM against existing single sample gene set testing methods, we measured the classification accuracy of each method (i.e. how well the method is able to highly rank cells that have inflated values for the genes in a specific set) on scRNA-seq data sets simulated according to the procedure outlined in the SI Methods. Use of classification accuracy vs. statistical power for the comparative evaluation had two motivations: (i) VAM is the only method in the comparison group that generates valid *P*-values and (ii) we envision VAM being used primarily as a means to rank order cells according to pathway activity rather than as a tool for cell-level statistical inference. Figure 2 illustrates the relative classification performance (as measured by the area under the receiver operating characteristic curve (AUC)) of VAM, GSVA (23), ssGSEA (24), and representative methods from the z-scoring and PCA-based categories (the technique of Lee *et al.* (28) for z-scoring and PLAGE (27) for PCA-based methods) across a range of sparsity, noise, effect size and set size values.

For each distinct combination of parameter values, 50 data sets were simulated according to the procedure outlined in the SI Methods and Figure 2 displays the average AUC for each method across these 50 data sets with error bars representing the standard error of the mean. The general trends in performance follow the expected trajectories, e.g. AUC values fall as sparsity or noise increase and AUC values increase as the effect size or set size increases. Importantly, the VAM method provides superior classification performance relative to the other evaluated methods across the full range of evaluated parameter values with the difference particularly pronounced for the sparsity and variance found in the PBMC scRNA-seq data.

### Computational efficiency

Table 1 displays the relative execution time of GSVA, ssGSEA and representative z-scoring and PCA-based methods as compared to VAM. Relative times are shown for the analysis of the simulated data sets (2000 cells and 500 genes) used to generate the classification results shown in Figure 2, for the analysis of the 3k cell PBMC scRNA-seq data set using the the BioCarta (C2.CP.BIOCARTA) collection from the Molecular Signatures Database (MSigDB) (34) (see the *Human PBMC analysis* Section for detailed results on the PBMC data set), for the analysis of the 11.8k cell mouse brain scRNA-seq data set using the MSigDB Gene Ontology biological process (C5.BP) pathway collection (see the *Mouse brain data analysis* Section for detailed results on the mouse brain data set), and for the analysis of the very large 242k cell Mouse Cell Atlas (MCA) (36) scRNA-seq data set using a collection comprised by one gene set for the first 50 genes. Since the R implementations of the comparison methods force the conversion of the gene expression matrix into a non-sparse format, these methods had to be executed on a subset of the MCA expression data containing just the 50 genes in the gene set to avoid memory limits. For the *z*-scoring and PCA-based methods, this subsetting does not impact the value of the generated gene-level scores. The GSVA and ssGSEA methods, however, require the full gene expression matrix for correct score generation and were therefore excluded from the analysis. For more details on the PBMC, mouse brain and MCA data sets and processing pipeline, please see the SI Methods.

**Table 1.** Relative execution time as compared to the VAM method on simulated scRNA-seq data, the PBMC scRNA-seq data set for MSigDB C2.CP.BIOCARTA collection and the mouse brain scRNA-seq data set for the MSigDB C5.BP collection

	Simulated	PBMC	Mouse brain	MCA
GSVA	426.29	26.23	3.60	—
ssGSEA	23.99	19.08	26.61	—
*z*-scoring	6.14	3.41	2.26	0.69
PCA	2.63	0.44	0.05	0.17

Although the simulation results reflect the average across a large number of simulated data sets, the real data results represent a single execution on the relevant scRNA-seq data. The VAM method had a much faster average execution on the simulated data set relative to the other methods with the difference particularly dramatic for the two most popular single sample methods, GSVA and ssGSEA. Although the relative performance values dropped on the real scRNA-seq data sets, with notable scaling efficiency for the PCA-based method, the relative efficiency of VAM compared to GSVA and ssGSEA on these real data sets was still large with the absolute difference in execution time substantial given the longer time taken by VAM on large data sets. For the very large MCA data set, only the VAM, *z*-scoring and PCA-based methods could be evaluated due to memory constraints. It should be noted that execution times can be highly variable and, for techniques like GSVA, strongly dependent on how effectively the logic can leverage parallel processing in the underlying architecture so users may encounter a wide range of relative performance values in practice.

### Human PBMC analysis

**Figure 3.**
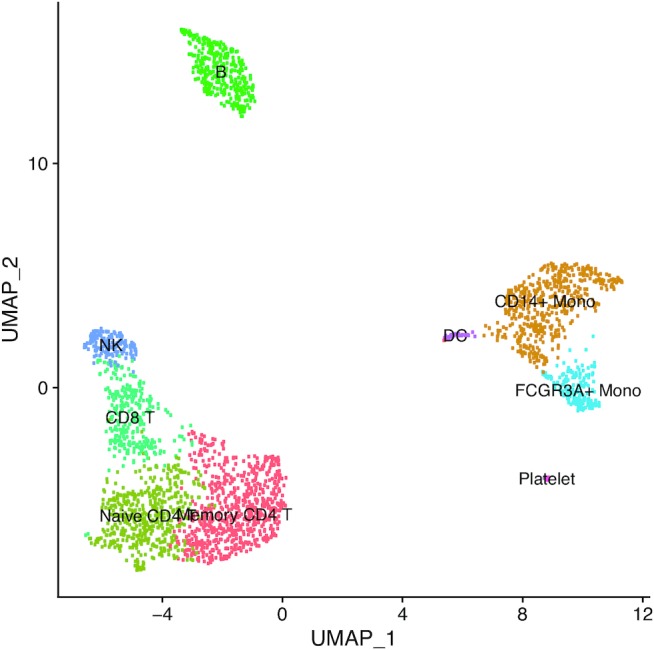
Projection of PBMC scRNA-seq data onto the first two UMAP dimensions. Each point in the plot represents one cell.

As detailed in the SI Methods, we applied the VAM method and comparison techniques to the 10× 2.7k human PBMC data set used in the Seurat Guided Clustering Tutorial (see the SI Methods for more details on this data set and the associated processing pipeline). Figure 3 is a reduced dimensional visualization of the 2638 cells remaining after quality control filtering. Cluster cell-type labels match the assignments in the Seurat Guided Clustering Tutorial. For this analysis, the cell-specific pathway scores were used to identify pathways with elevated activity within cell-type specific clusters. As an illustrative example, we highlight the results for the B cell cluster. Table 2 lists the five MSigDB BioCarta pathways most significantly up-regulated in the B cell cluster according to a Wilcoxon rank sum test applied to the cell-specific scores computed by VAM and other comparison methods. All of the evaluated methods correctly associate B cell-related pathways with the B cell cluster, which is not surprising given the very distinct transcriptomic profile of B cells. While all of the methods offer similar classification performance in this scenario, VAM still has the benefits of low computational cost and support for cell-level inference. For more complex cell populations, e.g. the mouse brain scRNA-seq data, VAM appears to offer superior classification performance relative to the other techniques.

**Table 2.** Top five BioCarta pathways found to have higher pathway activity scores in the B cell cluster relative to other cells in the PBMC data set according to a Wilcoxon rank sum test. Pathways are ordered according to *P*-value from Wilcoxon test. The columns reflect the method used to compute the cell-specific pathway scores.

VAM	GSVA	ssGSEA	*z*-scoring	PCA
IL5	CTCF	CTCF	IL5	BBCELL
BBCELL	BBCELL	ASBCELL	BBCELL	ASBCELL
ASBCELL	ASBCELL	BBCELL	BLYMPHOCYTE	TCRA
BLYMPHOCYTE	TH1TH2	IL5	MHC	CSK
INFLAM	IL5	TH1TH2	CTCF	TH1TH2

**Figure 4.**
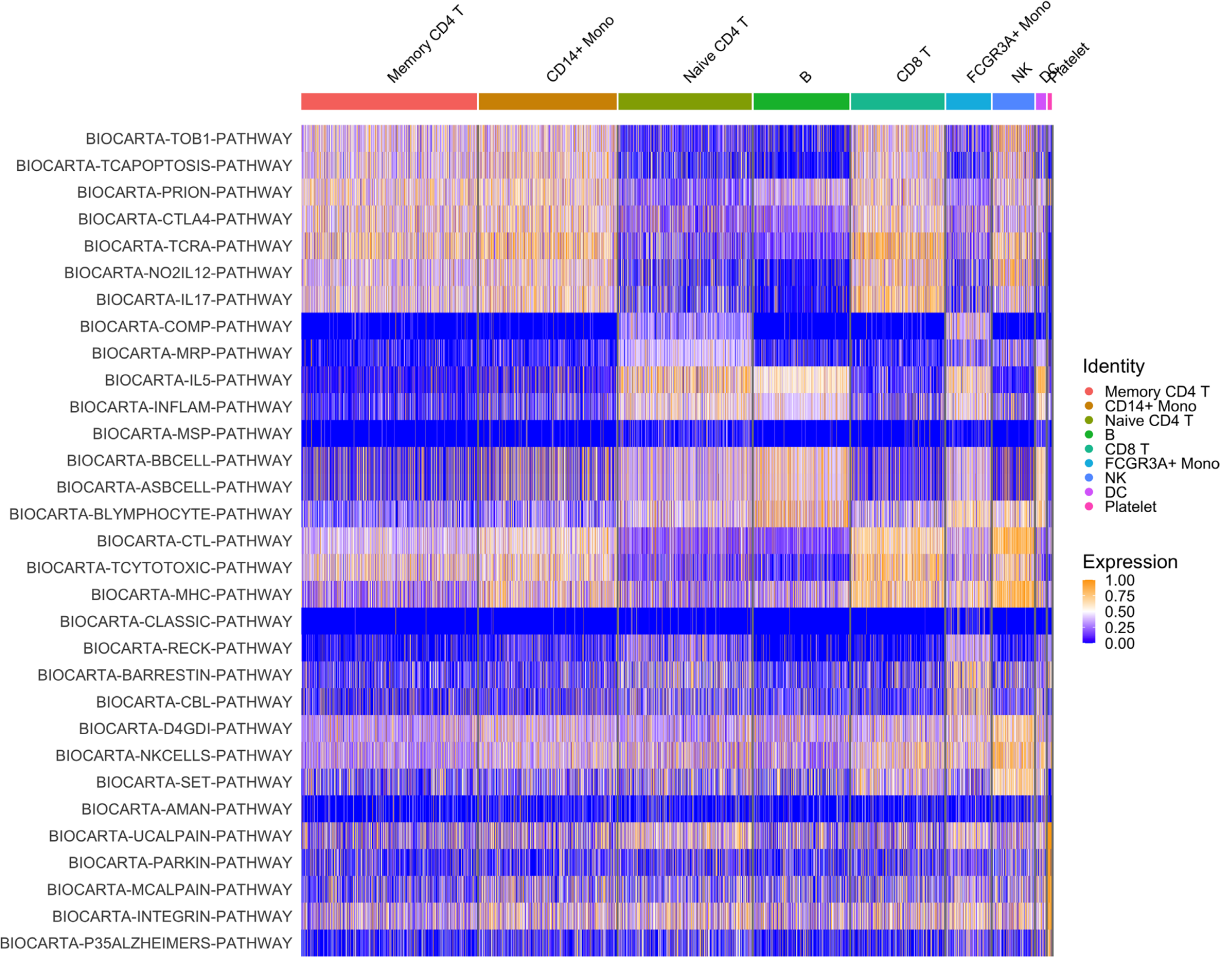
Heatmap visualization of the VAM generated cell-specific scores for the top five BioCarta pathways most significantly enriched in each cluster of the PBMC scRNA-seq data according to a Wilcoxon rank sum test on the VAM scores. Note that gene sets only appear once in the heatmap even if they are among the top five sets for multiple clusters.

A important use for the cell-specific scores generated by VAM is the visualization of pathway activity across all cells profiled in a given scRNA-seq data set. Figure 4 illustrates such a visualization for the four BioCarta pathways most significantly up-regulated in each cell type cluster according to cell-specific scores generated by the VAM method. This type of visualization provides important information regarding the range of pathway activity across all profiled cells, e.g. IL-5 activity is also up-regulated in monocytes. The VAM scores can also be visualized in a reduced dimensional space generated via a technique like UMAP (see Supplementary Figure S3 for an example).

### Mouse brain cell analysis

**Figure 5.**
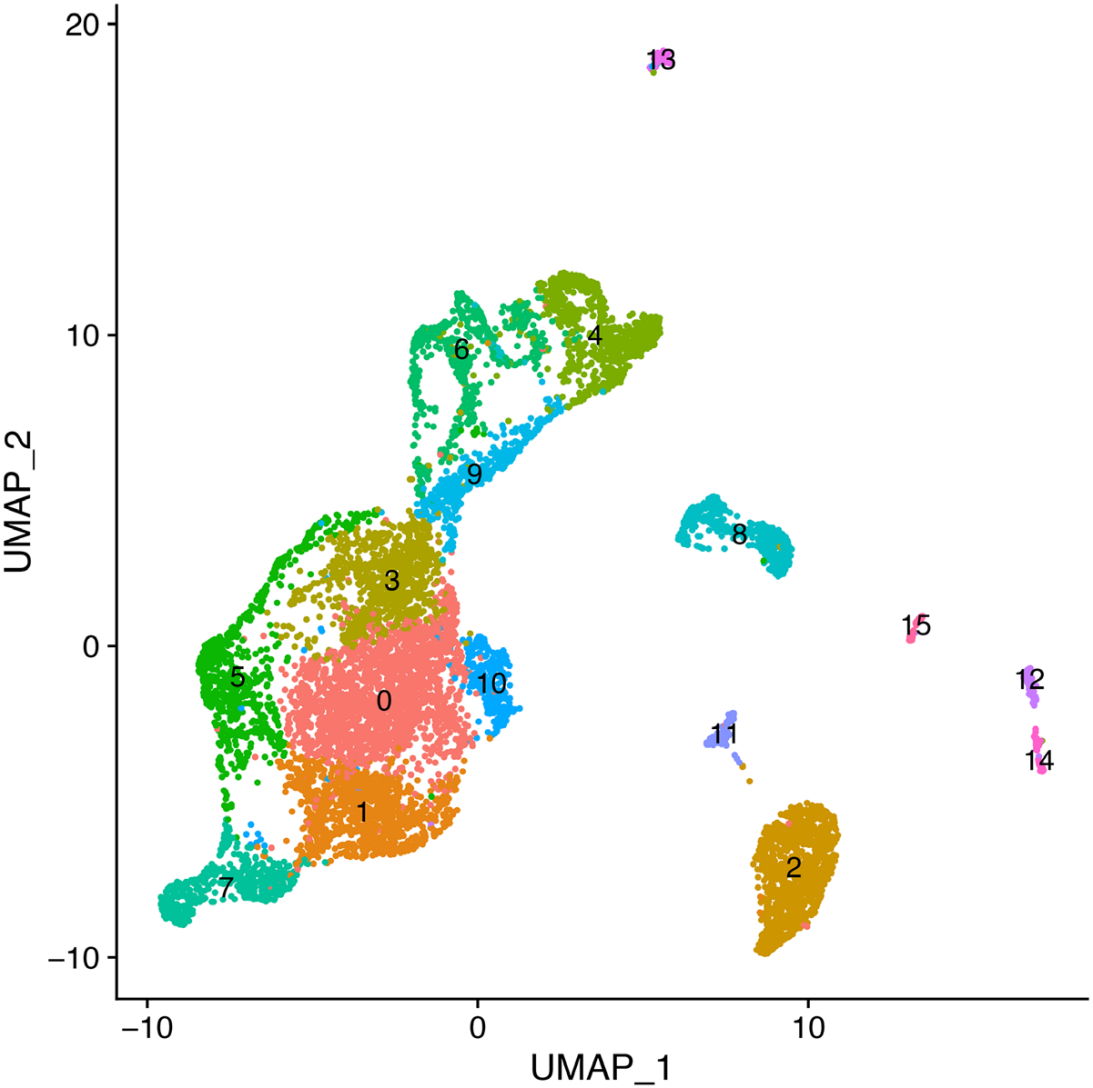
Projection of mouse brain scRNA-seq data onto the first two UMAP dimensions. Cells are labeled according to the output from unsupervised clustering.

As detailed in the SI Methods, we applied the VAM method and comparison techniques to the 10x 11.8k mouse brain scRNA-seq data set. For this example, we used the SCTransform normalization technique instead of log-normalization and explored a much larger pathway collection (the MSigDB Gene Ontology biological process (C5.BP) collection with 6097 gene sets after size-based filtering). Figure 5 is a reduced dimensional visualization of the 9320 cells remaining after quality control filtering with cells labeled according to the output from unsupervised clustering. Similar to the PBMC analysis, the cell-specific pathway scores were used to identify pathways with elevated activity within specific clusters.

We highlight the results for cluster 4, which appears to represent glial cells including a population of astrocytes, a glial cell subtype. Table 3 lists the five MSigDBC C5.BP gene sets most significantly up-regulated in cluster 4 according to a Wilcoxon rank sum test applied to the cell-specific scores computed by VAM and other comparison methods.

**Table 3.** Top five Gene Ontology Biological Process gene sets (from MSigDB C5.BP collection) found to have higher pathway activity scores in cluster 4 relative to other cells in the mouse brain data set according to a Wilcoxon rank sum test. Gene sets are ordered according to *P*-value from Wilcoxon test

VAM
GLIAL-CELL-DIFFERENTIATION
LEPTIN-MEDIATED-SIGNALING-PATHWAY
CHOLESTEROL-CATABOLIC-PROCESS
ASTROCYTE-DIFFERENTIATION
GLIAL-CELL-DEVELOPMENT
GSVA
POSITIVE-REGULATION-OF-EXTRACELLULAR-MATRIX...
POSITIVE-REGULATION-OF-POSTSYNAPTIC-MEMBRA...
GLIAL-CELL-FATE-COMMITMENT
CHOLESTEROL-CATABOLIC-PROCESS
NOTOCHORD-DEVELOPMENT
ssGSEA
STRESS-RESPONSE-TO-METAL-ION
POSITIVE-REGULATION-OF-EXTRACELLULAR-MATRIX...
CHOLESTEROL-CATABOLIC-PROCESS
GLIAL-CELL-FATE-COMMITMENT
REGULATION-OF-EXTRACELLULAR-MATRIX-ASSEMBLY
z-scoring
REGULATION-OF-EXTRACELLULAR-MATRIX-ASSEMBLY
REGULATION-OF-GROWTH-RATE
ADENOHYPOPHYSIS-DEVELOPMENT
PROSTATE-GLAND-MORPHOGENESIS
STRESS-RESPONSE-TO-METAL-ION
PCA
CELLULAR-RESPONSE-TO-COPPER-ION
RESPONSE-TO-ZINC-ION
CELLULAR-RESPONSE-TO-CADMIUM-ION
PROSTATE-GLAND-MORPHOGENESIS
RESPONSE-TO-COPPER-ION

**Figure 6.**
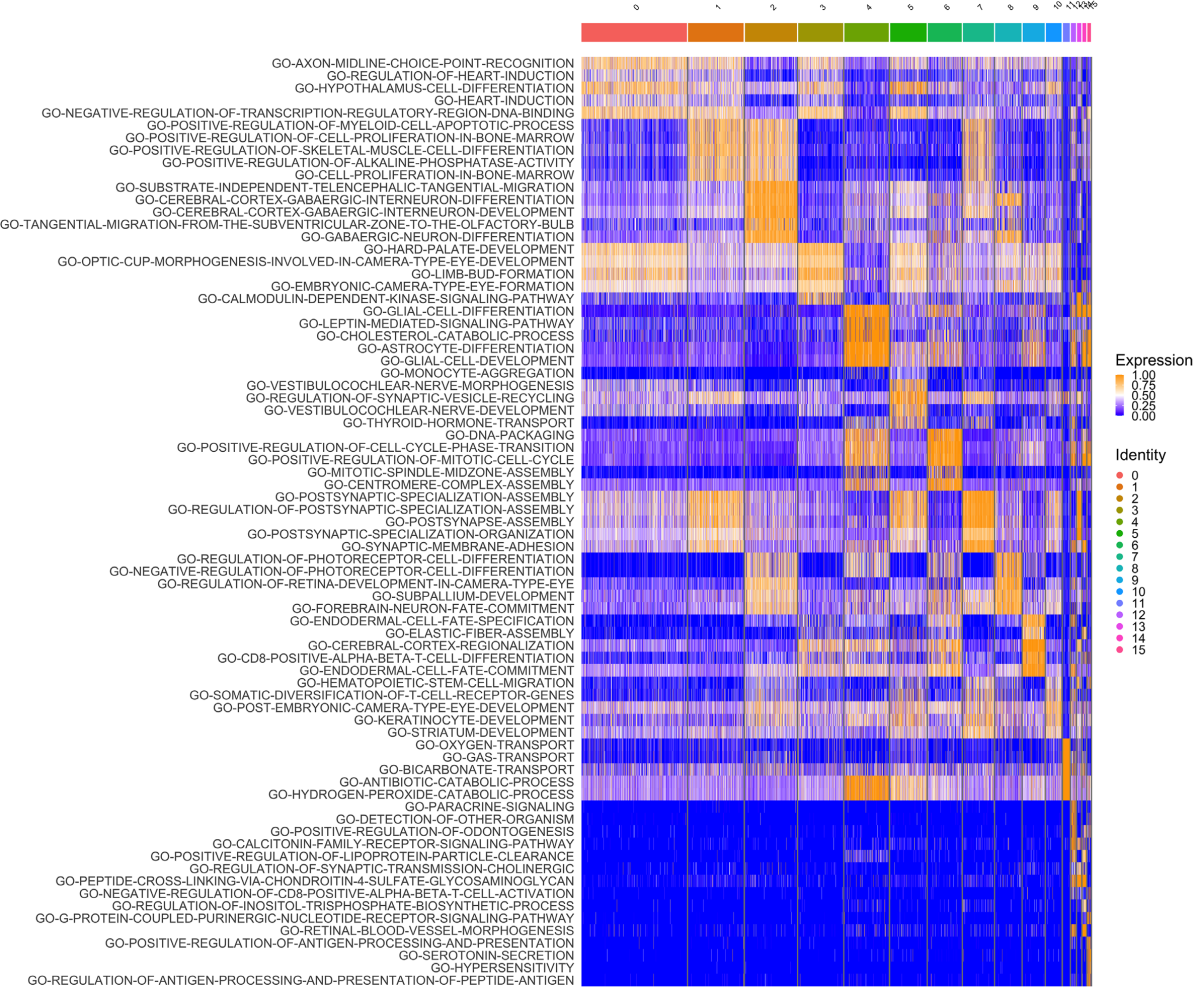
Heatmap visualization of the VAM generated cell-specific scores for the top five MSigDB C5.BP gene sets most significantly enriched in each cluster of the mouse brain scRNA-seq data (as seen in Figure 5) according to a Wilcoxon rank sum test on the VAM scores. Note that gene sets only appear once in the heatmap even if they are among the top five sets for multiple clusters.

As seen in Table 3, VAM clearly associates this cluster with glial cells with *GLIAL-CELL-DIFFERENTIATION* the top ranked set and both *ASTROCYTE-DIFFERENTIATION* and *GLIAL-CELL-DEVELOPMENT* also in the top five list. Figure 6 is a heatmap illustration of the VAM scores for the top five pathways in each cluster. A visualization of the VAM scores for the top four gene sets up-regulated in cluster 4 in the space of the first two UMAP dimensions can be found in Supplementary Figure S4. By contrast, neither the z-scoring nor PCA-based methods included glial cell-related sets in the top five and ssGSEA and GSVA each only identified one, *GLIAL-CELL-FATE-COMMITMENT*. None of these other methods identified an astrocyte-related gene set within the top five. Although it is not possible to say with certainty that cluster 4 captures the glial (and potentially astrocyte-specific) sub-population in this scRNA-seq data, the top five most significantly up-regulated genes in cluster 4 according to a Wilcoxon test on the SCTransform-corrected counts all have a known association with astrocytes: Dbi (37), Ptn (38), Tubb4b (39), Hopx (40), Igfbp2 (41).

**Figure 7.**
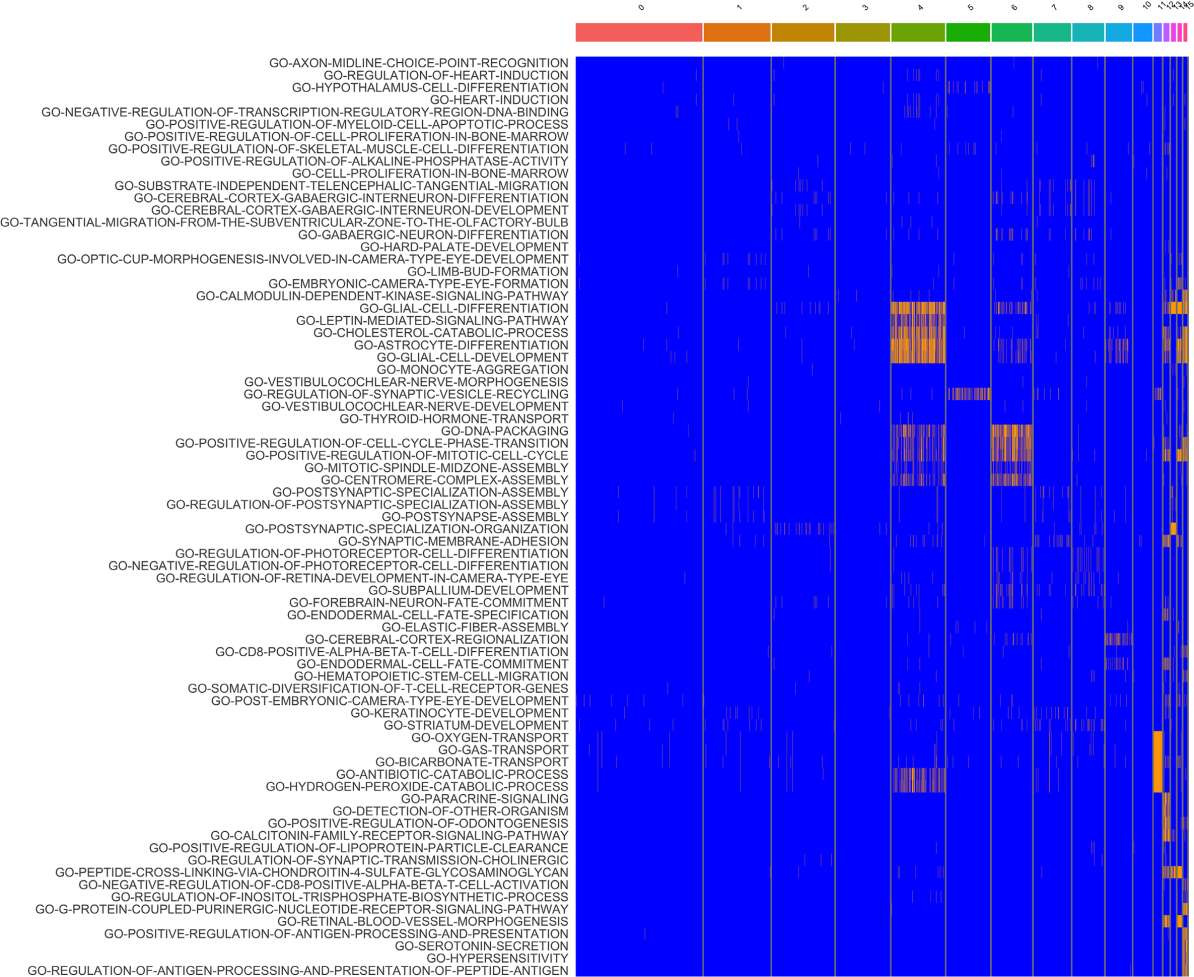
Heatmap visualization of VAM score statistical significance for the top five MSigDB C5.BP gene sets most enriched in each cluster of the mouse brain scRNA-seq data (as seen in Figure 5). Scores that are significant at an FDR of 0.1 are shown in orange and non-significant scores are shown in blue. Note that gene sets only appear once in the heatmap even if they are among the top five sets for multiple clusters.

The fact that the VAM scores can be easily converted into *P*-values according to the *H*_0_ of uncorrelated technical noise enables the use of cell-level inference for this example. Specifically, if we treat all ∼57 million computed scores as a family of hypotheses, ∼1.9% of the scores are significant at a false discovery rate (FDR) of 0.1 as computed using the Benjamini and Hochberg method (42). These inferential results are visualized in Figure 7, which indicates that the glial cell signature is statistically significant for most cells in cluster 4 according hypothesis tests on the VAM scores. Given the very large size of the family of tested hypotheses, this result provide strong support for the glial cell association with cluster 4. For scenarios where cell-level inference is the primary goal, statistical power can be greatly increased by using a more targeted collection of gene sets, e.g. just the signatures of cell types expected in the analyzed tissue. It is important to note that this type of cell-level inference is not supported by any other existing single sample gene set testing methods.

## CONCLUSION

Single cell RNA-sequencing is a powerful experimental tool for exploring the biology of heterogeneous cell populations. The significant sparsity and technical noise associated with scRNA-seq data, however, makes statistical analysis challenging, especially for tests conducted on the level of individual genes. One promising approach for addressing the statistical challenges of scRNA-seq data is gene set testing or pathway analysis, a hypotheses aggregation technique that can mitigate the issues of sparsity and technical noise to improve power, replication and interpretability. The class of single sample gene set testing methods, which transform a cell-by-gene matrix into a cell-by-pathway matrix, is particular effective for single cell analyses since it enables the full range of standard downstream processing (visualization, clustering, differential expression testing etc.) to be performed on the pathway-level rather than on the gene-level. Unfortunately, almost all existing single sample gene set testing methods were designed for the analysis of bulk tissue gene expression data, which is non-sparse and, compared to scRNA-seq data, has a small sample size and limited technical noise.

To remedy the lack of effective single sample gene set testing methods for scRNA-seq data, we developed the variance-adjusted Mahalanobis (VAM) method, a novel modification of the standard Mahalanobis multivariate distance measure that generates cell-specific pathways scores which account for the inflated noise and sparsity of scRNA-seq data. Although we expect the scores generated by VAM to be primarily used in contexts that do not assume a specific statistical model, e.g. as predictor variables, the fact that the distribution of the VAM-generated scores has an accurate gamma approximation under the null of uncorrelated technical noise enables inference regarding pathway activity for individual cells. As demonstrated on both simulated and real scRNA-seq data, the VAM method provides superior classification performance at low computational cost relative to existing single sample techniques. The utility of VAM is also aided by direct integration with the popular Seurat framework, which makes it easy to incorporate VAM into existing scRNA-seq analysis pipelines. These features combine to make the VAM method an effective and practical tool for the visualization and statistical analysis of scRNA-seq data.

## Supplementary Material

gkaa582_Supplemental_File
