# Supplementary material for: Variance-adjusted Mahalanobis (VAM): a fast and accurate method for cell-specific gene set scoring
Source: Nucleic Acids Res. 2020 Jul 7;48(16):e94. doi: 10.1093/nar/gkaa582 (PMC7498348; doi:10.1093/nar/gkaa582)
Supplement: gkaa582_Supplemental_File [file gkaa582_supplemental_file.pdf]

# Variance-adjusted Mahalanobis (VAM): a fast and accurate method for cell-specific gene set scoring
# Supplemental Information

H. Robert Frost *

## Contents

**1 Supplemental Methods** **2**
1.1 VAM-Seurat integration . . . . . . . . . . . . . . . . . . . . . . . . . . . . . . 2
1.1.1 Integration for log-normalization . . . . . . . . . . . . . . . . . . . . . . . 2
1.1.2 Integration for SCTransform . . . . . . . . . . . . . . . . . . . . . . . . . 2
1.1.3 Integration for other frameworks . . . . . . . . . . . . . . . . . . . . . . . 2
1.2 Simulation study design . . . . . . . . . . . . . . . . . . . . . . . . . . . . . . 3
1.3 Real data analysis design . . . . . . . . . . . . . . . . . . . . . . . . . . . . . 3

**2 Supplemental Results** **5**
2.1 Type I error control and power . . . . . . . . . . . . . . . . . . . . . . . . . . 5
2.2 Human PBMC analysis . . . . . . . . . . . . . . . . . . . . . . . . . . . . . . 6
2.3 Mouse brain cell analysis . . . . . . . . . . . . . . . . . . . . . . . . . . . . . 7

## List of Figures

S1 Distribution of non-zero log-normalized counts from the PBMC scRNA-seq data set. Both the kernel density estimate and associated log-normal density are displayed. . . 3
S2 Estimated statistical power for the VAM method on simulated scRNA-seq data at different effect size values. . . . . . . . . . . . . . . . . . . . . . . . . . . . . . 5
S3 Visualization of the VAM generated cell-specific scores for the four BioCarta pathways most significantly enriched in the B cell cluster according to a Wilcoxon rank sum test on the VAM scores. . . . . . . . . . . . . . . . . . . . . . . . . . . . . 6
S4 Visualization of the VAM generated cell-specific scores for the MSigDB C5.BP gene sets most significantly enriched in cluster 4 of the mouse brain scRNA-seq data (as seen in Figure ??) according to a Wilcoxon rank sum test on the VAM scores. . . . . 7

*rob.frost@dartmouth.edu, Department of Biomedical Data Science, Geisel School of Medicine, Dartmouth College, Hanover, NH 03755

# 1 Supplemental Methods

## 1.1 VAM-Seurat integration

The VAM implementation supports direct integration with the Seurat framework [1] with the integration details varying based on whether Seurat log-normalization is followed by variable feature detection using a mean/variance trend or the SCTransform [2] method is used to perform both normalization and variable feature detection. The **S** matrix generated by VAM is saved as a new Seurat assay, which enables the visualization and further analysis of these cell-specific pathway scores using Seurat framework, e.g., the *FeaturePlot()* and *FindMarkers()* functions.

### 1.1.1 Integration for log-normalization

The Seurat log-normalization method implemented by the *NormalizeData()* R function starts by dividing the unique molecular identifier (UMI) counts for each gene in a specific cell by the sum of the UMI counts for all genes measured in the cell and multiplying this ratio by the scale factor $1 \times 10^6$. The normalized scRNA-seq values are then generating by taking the natural log of this relative value plus 1. When log-normalization is used, variable features are detected using the *FindVariableFeatures()* function, which fits a non-linear trend to the log scale variance/mean relationship (the Seurat *vst* method). The estimated trend models the expected technical variance based on mean gene expression; observed variance values above this expected trend reflect biological variance. Given this trend, the proportion of technical variance is computed as ratio of the expected technical variance to the observed variance. Note that it is possible for this ratio to be greater than 1 if the observed variance is less that the expected variance. In this scenario, VAM sets the **X** matrix to the log-normalized values and the technical variance vector $\hat{\boldsymbol{\sigma}}^2_{\text{tech}}$ is computed as the product of the variance of the normalized counts and the proportion of technical variance as estimated by the *vst* method.

### 1.1.2 Integration for SCTransform

The Seurat SCTransform normalization method [2] fits a regularized negative binomial regression model on the UMI counts for each gene using approximate cell sequencing depth as a dependent variable. The Pearson residuals from these regression models capture the biological component of the scRNA-seq data and should have a mean of 0 and variance of 1 if expression is due solely to technical noise. The reciprocal of the Pearson residual variance therefore estimates the proportion of technical variance. In this scenario, VAM sets the **X** matrix to 1 plus the natural log of the corrected UMI counts (i.e., counts that have been adjusted using the Pearson residuals to reflect the counts that would be observed if all cells had the same sequencing depth) and the technical variance vector $\hat{\boldsymbol{\sigma}}^2_{\text{tech}}$ is computed as the ratio of the variance of corrected counts in **X** and the variance of the Pearson residuals.

### 1.1.3 Integration for other frameworks

If VAM is used with other frameworks, an appropriate method must be used to decompose the measured variance of each gene into technical and biological components. Other popular scRNA-seq frameworks like Scater [3] support this decomposition using an approach similar to that taken by Seurat, i.e, fitting a nonlinear mean-dependent trend to the unnormalized sample variances. Users of other frameworks are also encouraged to explore existing Seurat support and guidance for

converted to/from those frameworks and Seurat:
https://satijalab.org/seurat/v3.1/conversion_vignette.html.

## 1.2 Simulation study design

To explore the statistical properties of the VAM method, we used simulated scRNA-seq data simulated to reflect the characteristics of the PBMC log-normalized data. To simulate normalized scRNA-seq data, i.e, the contents of **X**, we took advantage of the fact that the non-zero log-normalized values in real scRNA-seq data sets can be effectively modeled by a log-normal distribution. Figure S1 illustrates this distributional approximation for the non-zero log-normalized counts from the peripheral blood mononuclear cell (PBMC) scRNA-seq data set (see Section 1.3 for more details on this data set). Based on this result, we simulated normalized scRNA-seq data under the $H_0$ of uncorrelated technical noise by first populating **X** for 2,000 cells and 500 genes with independent log-normal RVs with mean and variance set to the sample estimates for the non-zero normalized counts in the PBMC scRNA-seq data. The generated **X** was then sparsified by setting a random selection of elements to 0, with the number of zero elements matching the desired sparsity level. Data sets simulated according to this procedure were used to generate Figure 1 in the main manuscript as well as the type I error control results.

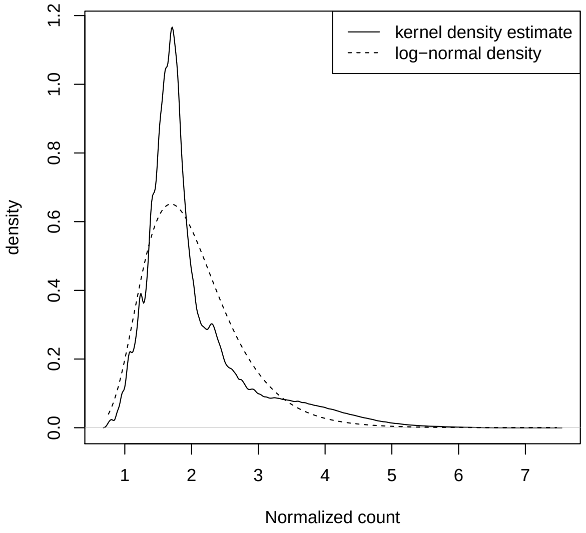


Figure S1: Distribution of non-zero log-normalized counts from the PBMC scRNA-seq data set. Both the kernel density estimate and associated log-normal density are displayed.

To assess power and classification performance, data sets simulated under $H_0$ were modified to elevate the normalized expression of genes in a hypothetical gene set of size 50 for the first 50 cells while preserving the overall sparsity level. The elevated values were computed by setting all non-zero counts in the original null data to log-normal RVs with a larger mean (the variance was set to the same value used to simulate the null data). Classification performance was assessed for a range of null variance, set size and inflated mean values.

## 1.3 Real data analysis design

To assess the performance of VAM on real scRNA-seq data, we analyzed three data sets that are freely available from 10x Genomics: the 2.7k human PBMC data set used in the Seurat Guided Clustering Tutorial [4], an 11.8k mouse brain cell data set generated on the combined cortex, hippocampus and sub ventricular zone of an E18 mouse [5], and the 242k cell Microwell-seq Mouse Cell Atlas (MCA) [6,7]. These data sets are representative of small, medium, and very large sized single cell experiments and capture transcriptomic measurements for heterogeneous cells populations for the two organisms (human and mouse) that comprise a large fraction of existing scRNA-seq data sets.

Preprocessing, quality control (QC), normalization and clustering of the PBMC data set followed the exact processing steps used in the Seurat Guided Clustering Tutorial. Specifically, the Seurat log-normalization method was used followed by application of the *vst* method for decom-

posing technical and biological variance. Preprocessing and QC of the PBMC data yielded an **X** matrix of normalized counts for 14,497 genes and 2,638 cells.

Processing of the mouse brain data followed similar quality control metrics (at least 200 features per cell, non-zero values in at least 10 cells for genes, proportion of mitochondrial reads less than 10% [8]) with Uniform Manifold Approximation and Projection (UMAP) [9] used for dimensionality reduction and clustering performed with Seurat's implementation of shared nearest neighbor (SNN) modularity optimization [10]. Normalization of the mouse brain data was performed using SCTransform rather than log-normalization to explore the performance of VAM for both of the supported Seurat normalization approaches. Preprocessing and QC of the mouse brain data yielded an **X** matrix of normalized counts for 32,850 genes and 9,320 cells.

For the VAM analysis of the PBMC and mouse brain scRNA-seq data sets, the gene set matrix **A** was populated using the C2.CP.BIOCARTA (BioCarta, 289 gene sets, 261 after filtering), and the C5.BP (Gene Ontology Biological Processes, 7,350 gene sets) collections from the version 7.0 of the Molecular Signatures Database (MSigDB) [11]. These MSigDB collections contain gene sets from three well known and widely used repositories of curated gene sets: BioCarta [11], and the biological process branch of the Gene Ontology [12]. Prior to running VAM, the Entrez gene IDs used by MSigDB were converted to Ensembl IDs using logic in the Bioconductor *org.Hs.eg.db* R package [13]. For analysis of the mouse brain data, the human Ensembl IDs were mapped to murine orthologs using logic in the *biomaRt* R package [14]. The **X** and **A** matrices were then filtered to only contain genes present in both matrices (13,714 genes for the PBMC data and 16,425 genes for the mouse brain data). Finally, the **A** matrix was filtered to remove all gene sets containing fewer than 5 or more than 200 members leaving a total of 261 gene sets for BioCarta and 6,097 gene sets for Gene Ontology Biological Processes). To determine enrichment of gene sets for specific scRNA-seq clusters, a Wilcoxon rank sum test was performed using the Seurat *FindMarkers()* method.

Processing of the MCA data followed the exact steps used in the Seurat Guided Clustering of the Microwell-seq Mouse Cell Atlas Tutorial [7]. Specifically, the Seurat log-normalization method was used followed by application of the *vst* method for decomposing technical and biological variance. Preprocessing and QC of the MCA data yielded an **X** matrix of normalized counts for 39,855 genes and 242,533 cells. Gene set analysis of the MCA data focused on the relative computational performance of VAM, ssGSEA, GSVA and the representative z-scoring the PCA-based techniques. For this purpose, a collection with a single gene set for the first 50 genes was analyzed. Because the R implementations of the comparison methods force the conversion of the gene expression matrix into a non-sparse format, these methods had to be executed on a subset of the expression data that contained just the first 50 genes to avoid memory limits. For the z-scoring and PCA-based methods, this subsetting does not impact the value of the generated gene-level scores. The GSVA and ssGSEA methods, however, require the full gene expression matrix for correct score generation and were therefore excluded from the MCA analysis.

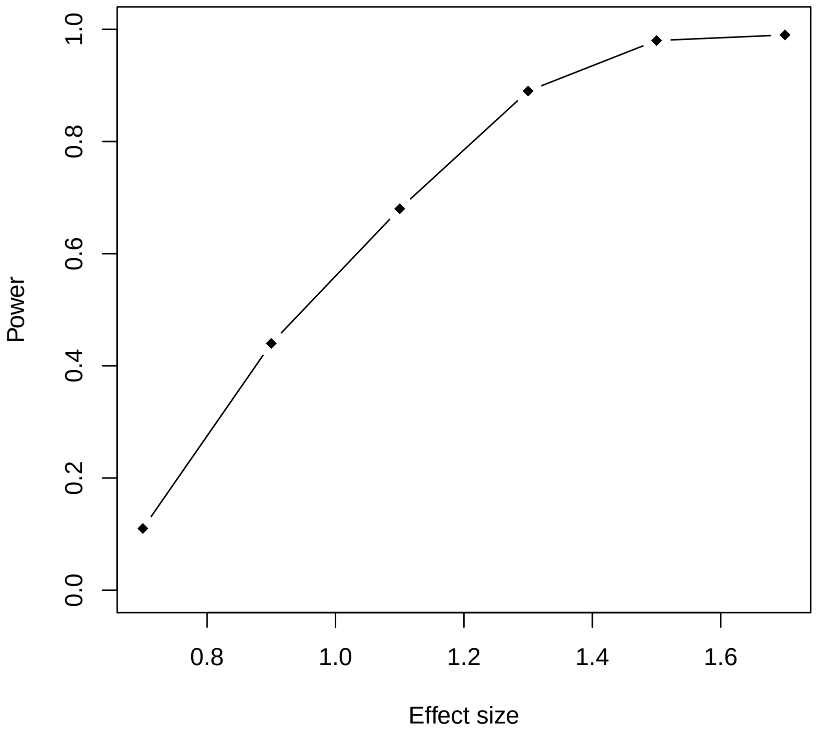


Figure S2: Estimated statistical power for the VAM method on simulated scRNA-seq data at different effect size values.

# 2 Supplemental Results

## 2.1 Type I error control and power

Figure S2 illustrates the power vs. effect size curve for the simulation design detailed Section 1.2. In this case, the estimated power values ranged from 0.11 for an inflated mean of 0.7 to 0.99 for an inflated mean of 1.7.

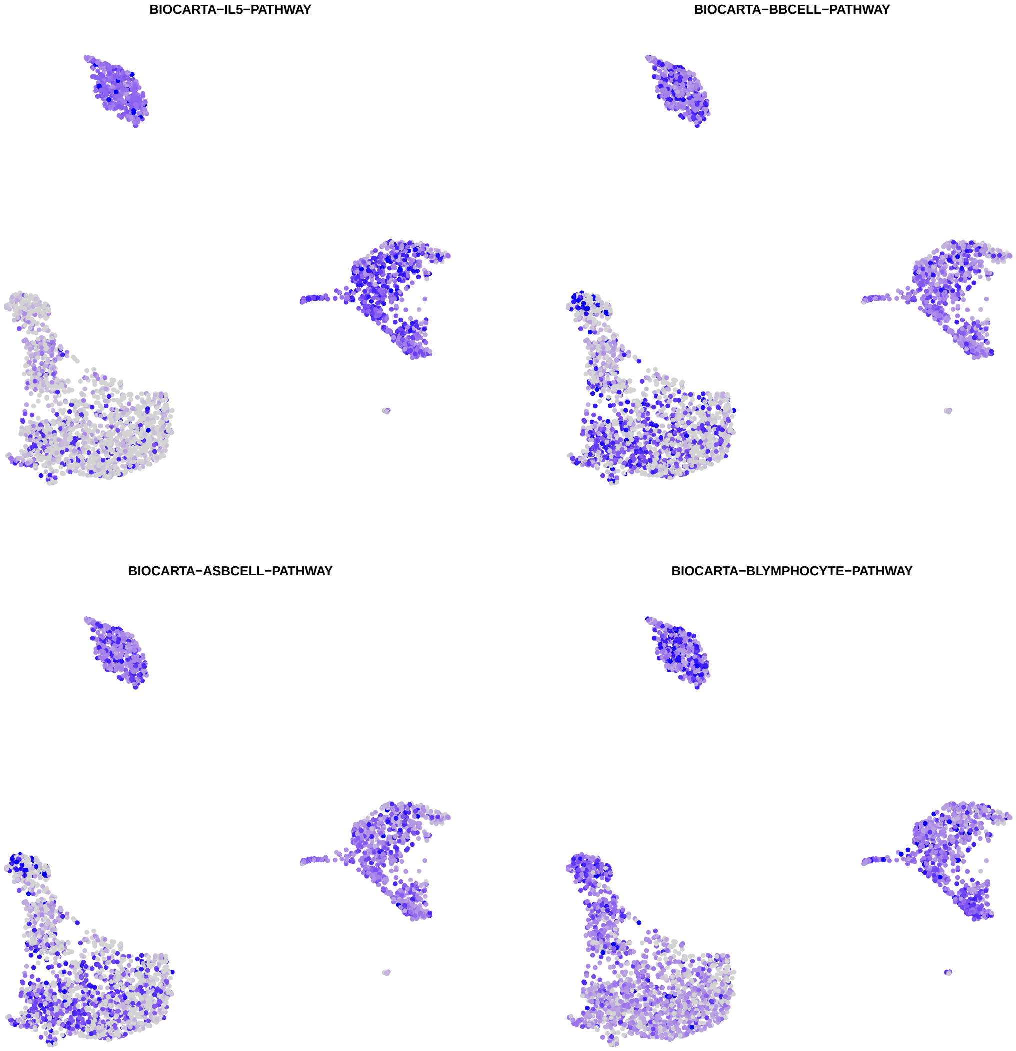


Figure S3: Visualization of the VAM generated cell-specific scores for the four BioCarta pathways most significantly enriched in the B cell cluster according to a Wilcoxon rank sum test on the VAM scores.

## 2.2 Human PBMC analysis

A important use for the cell-specific scores generated by VAM is the visualization of pathway activity across all cells profiled in a given scRNA-seq data set. Figure S3 illustrates such a visualization in the first two UMAP dimensions for the four BioCarta pathways most significantly up-regulated in the B cell cluster according to cell-specific scores generated by the VAM method. These types of visualizations provides important information regarding the range of pathway activity across all profiled cells, e.g., IL-5 activity is also up-regulated in monocytes.

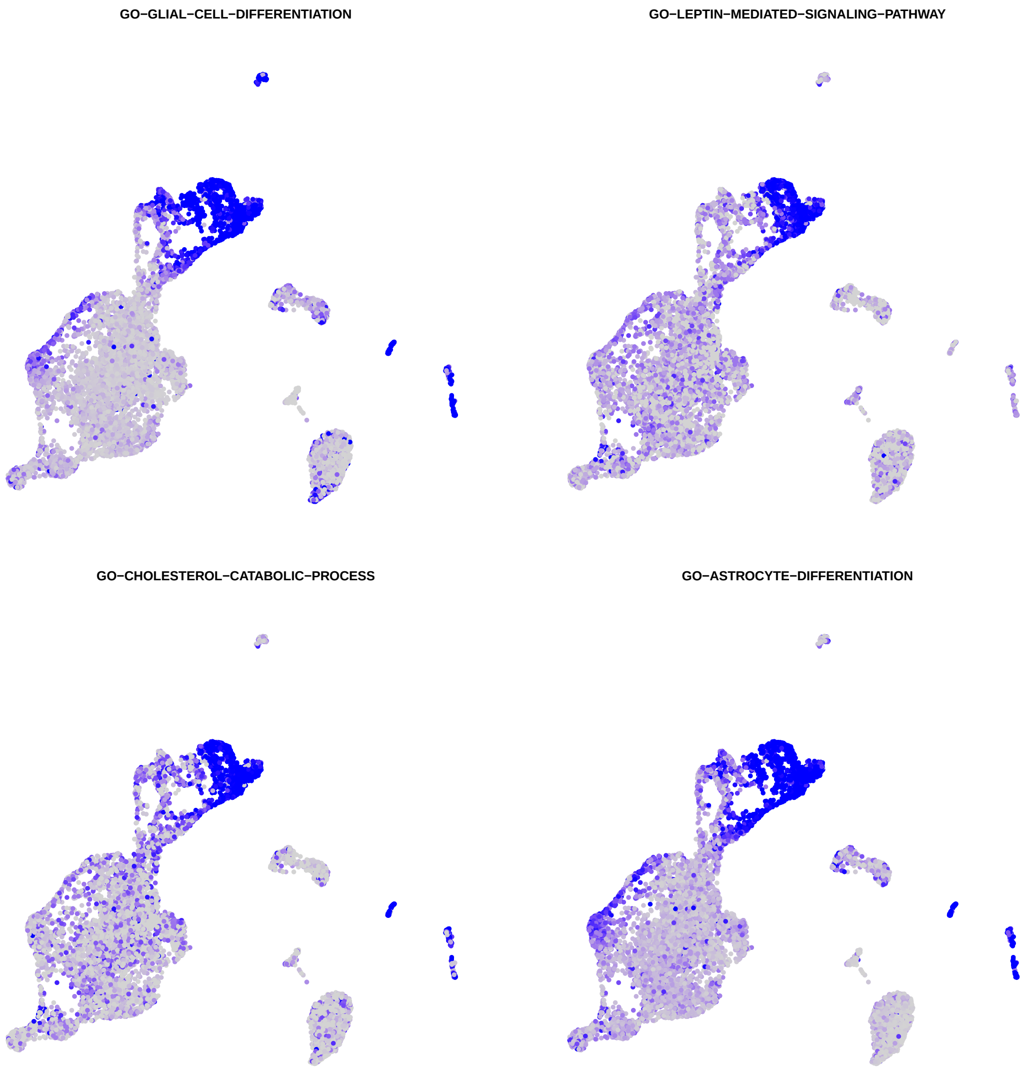


Figure S4: Visualization of the VAM generated cell-specific scores for the MSigDB C5.BP gene sets most significantly enriched in cluster 4 of the mouse brain scRNA-seq data (as seen in Figure **??**) according to a Wilcoxon rank sum test on the VAM scores.

## 2.3 Mouse brain cell analysis

Figure S4 provides a visualization in the space of the first two UMAP dimensions of the VAM-generated scores for the top four gene sets up-regulated in cluster 4 of the mouse neuron scRNA-seq data (see the main manuscript for a visualization of the clusters).

## References


[1] Stuart, T., Butler, A., Hoffman, P., Hafemeister, C., Papalexi, E., Mauck, W.M. 3rd, Hao, Y., Stoeckius, M., Smibert, P., Satija, R.: Comprehensive integration of single-cell data. Cell **177**(7), 1888–190221 (2019). doi:10.1016/j.cell.2019.05.031

[2] Hafemeister, C., Satija, R.: Normalization and variance stabilization of single-cell rna-seq data using regularized negative binomial regression. Genome Biol **20**(1), 296 (2019). doi:10.1186/s13059-019-1874-1

[3] McCarthy, D.J., Campbell, K.R., Lun, A.T.L., Wills, Q.F.: Scater: pre-processing, quality control, normalization and visualization of single-cell rna-seq data in r. Bioinformatics **33**(8), 1179–1186 (2017). doi:10.1093/bioinformatics/btw777

[4] Seurat: Seurat Guided Clutering Tutorial. `https://satijalab.org/seurat/v3.1/pbmc3k_tutorial.html`. Accessed: 2020-02-10

[5] 10x Genomics: 10k Brain Cells from an E18 Mouse (v3 chemistry). `https://support.10xgenomics.com/single-cell-gene-expression/datasets/3.0.0/neuron_10k_v3?` Accessed: 2020-02-10

[6] Han, X., Wang, R., Zhou, Y., Fei, L., Sun, H., Lai, S., Saadatpour, A., Zhou, Z., Chen, H., Ye, F., Huang, D., Xu, Y., Huang, W., Jiang, M., Jiang, X., Mao, J., Chen, Y., Lu, C., Xie, J., Fang, Q., Wang, Y., Yue, R., Li, T., Huang, H., Orkin, S.H., Yuan, G.-C., Chen, M., Guo, G.: Mapping the mouse cell atlas by microwell-seq. Cell **173**(5), 1307 (2018). doi:10.1016/j.cell.2018.05.012

[7] Seurat: Seurat Guided Clustering of the Microwell-seq Mouse Cell Atlas. `https://satijalab.org/seurat/v3.0/mca.html`. Accessed: 2020-06-10

[8] Carter, R.A., Bihannic, L., Rosencrance, C., Hadley, J.L., Tong, Y., Phoenix, T.N., Natarajan, S., Easton, J., Northcott, P.A., Gawad, C.: A single-cell transcriptional atlas of the developing murine cerebellum. Curr Biol **28**(18), 2910–29202 (2018). doi:10.1016/j.cub.2018.07.062

[9] McInnes, L., Healy, J., Melville, J.: UMAP: Uniform Manifold Approximation and Projection for Dimension Reduction (2018). 1802.03426

[10] Waltman, L., van Eck, N.J.: A smart local moving algorithm for large-scale modularity-based community detection. The European Physical Journal B **86**(11) (2013). doi:10.1140/epjb/e2013-40829-0

[11] Liberzon, A., Subramanian, A., Pinchback, R., Thorvaldsdóttir, H., Tamayo, P., Mesirov, J.P.: Molecular signatures database (msigdb) 3.0. Bioinformatics **27**(12), 1739–40 (2011). doi:10.1093/bioinformatics/btr260

[12] Gene Ontology Consortium: The gene ontology in 2010: extensions and refinements. Nucleic Acids Res **38**(Database issue), 331–5 (2010). doi:10.1093/nar/gkp1018

[13] Carlson, M.: org.Hs.eg.db: Genome Wide Annotation for Human. (2019). R package version 3.8.2

[14] Durinck, S., Spellman, P.T., Birney, E., Huber, W.: Mapping identifiers for the integration of genomic datasets with the r/bioconductor package biomart. Nat Protoc **4**(8), 1184–91 (2009). doi:10.1038/nprot.2009.97
